# Evaluation of retinal sensitivity and microstructure in areas of capillary nonperfusion of eyes with branch retinal vein occlusion

**DOI:** 10.1186/s12886-021-02089-w

**Published:** 2021-09-10

**Authors:** Puying Wei, Chenchen Liu, Yanzhen Zhang, Liu Yang

**Affiliations:** Department of Ophthalmology, Peking University First Hospital, No. 8 Xishiku Street, Xicheng District, 100034 Beijing, China

**Keywords:** Microperimetry, Optical coherence tomography angiography, Retinal vein occlusion, Disorganization of retinal inner layer

## Abstract

**Background:**

To evaluate macular microstructure alterations in the parafoveal nonperfusion areas of eyes with branch retinal vein occlusions (BRVO), and to investigate their impact on retinal sensitivity.

**Methods:**

This was a cross-sectional study including thirteen BRVO patients with parafoveal capillary nonperfusion areas (NPA). Multiple modalities including microperimetry, optical coherence tomography angiography, and optical coherence tomography were performed to measure retinal sensitivity and thickness, and to identify the microstructure changes and perfusion status.

**Results:**

The retinal sensitivity and thickness in the NPA were significantly lower than those in the perfusion areas (PA) (*P* = 0.001, *P* = 0.003). Microstructure changes, including disorganization of the retinal inner layers (DRIL), disruption of the outer retinal layers, and cysts were more frequently observed in NPA (*P* = 0.002, *P* = 0.018, *P* = 0.068). Within NPA, the retinal sensitivity of areas with DRIL, and outer retinal layers disruption was significantly lower than that of the areas without DRIL (*P* = 0.016), and with intact outer retinal layers (*P* < 0.001), respectively. 1dB increase in retinal sensitivity was correlated with 2.2 μm (95 % confidence interval, 1.71–2.7) increase of the thickness (*P* < 0.001). The retinal sensitivity was significantly lower at points with both DRIL and outer retinal layers disruption than at the points with DRIL or outer retina layers disruption alone (*P* = 0.001, *P* = 0.001).

**Conclusions:**

Alterations in the macular microstructure are associated with ischemia, especially DRIL. DRIL and outer retinal layers disruption are imaging features that have important implications for local retinal sensitivity in the ischemic areas, and where the microstructure of both inner and outer retinal layers is disrupted the function is further destructed.

## Background

Branch retinal vein occlusion (BRVO) is one of the most common primary vascular diseases of retina, and is often associated with vision loss. Macular edema (ME) is the dominant cause of decreased visual acuity in most patients, and macular ischemia also contributes to poor visual prognoses [1]. The visual prognosis of BRVO has radically improved by the recent introduction of anti-vascular endothelial growth factor (VEGF) treatment [2, 3]. However, even with successful treatments that utilize anti-VEGF for macular edema, the capillary dropout area before treatment may not undergo reperfusion [4, 5].

Polat et al. reported that ischemic retinal vein occlusion (RVO) caused greater macular edema and disruption in macular microstructures compared to nonischemic RVO, especially in terms of disorganization of the retinal inner layers (DRIL) and disrupted external limiting membrane (ELM) [6]. DRIL is an imaging feature of spectral domain-optical coherence tomography (SD-OCT) that has become recognized as a sign of impaired visual function [7–12], and a reliable predictor of capillary nonperfusion areas [13, 14].

In recent years, as microperimetry (MP) examination technology has developed, built-in eye-tracking systems was used, which enabled the accurate evaluation of macular retina sensitivity, and assessed precise associations between retinal function and localized lesions [15]. For instance, it has been reported that retinal sensitivity is remarkably reduced within nonperfused areas in eyes with BRVO [16, 17]; while to date, no study has examined the sensitivity of retina with DRIL.

The objectives of this investigation were to evaluate macular microstructure alterations, such as DRIL, of parafoveal nonperfusion areas in eyes with BRVO, and to investigate their impact on retinal sensitivity by adopting multiple modalities, including SD-OCT, optical coherence tomography angiography (OCTA), and MP-3.

## Methods

### Subjects

This study complied with the principles of the Declaration of Helsinki, and informed consent was acquired from all participants. This study was approved by the National Unit of the Clinical Trial Ethics Committee, Peking University First Hospital.

Between November 2020 to January 2021, this cross-sectional and observational study enrolled 13 BRVO patients with parafoveal capillary nonperfusion area (NPA) [17], who were examined at the Department of Ophthalmology at Peking University First Hospital. All eyes had shown marked ME, and/or serous retinal detachment, and/or retinal hemorrhage that involved the fovea during the initial visit, with a central retinal thickness (CRT) greater than 300 μm. All eyes showed complete or partial resolution of their ME at the time of inclusion in the current study, with a CRT lower than 300 μm, and a best-corrected visual acuity (BCVA) better than 35 Early Treatment Diabetic Retinopathy Study (ETDRS) letters. Patients with a less than 5-month history of RVO-ME, media opacities, co-existing ocular diseases (e.g., epiretinal membranes, glaucoma, diabetic retinopathy, age-related macular degeneration, epiretinal membranes), spherical equivalent > + 3.0D or < − 6.0D, serious retinal detachment, retinal hemorrhage, macular grid photocoagulation, poor-quality images, or without parafoveal NPAs were excluded, along with patients who had undergone surgical treatment, except for anti-VEGF intravitreal therapy, more than 2 months ago.

All eyes were diagnosed and their subtypes were determined via comprehensive ophthalmologic examinations. Intravitreal anti-VEGF treatment was the first-line treatment for ME. A sub-Tenon’s capsule injection of triamcinolone acetonide or observation were chosen for cost reasons. Scatter laser photocoagulation was performed for retinal neovascularization and/or NPAs > 5PD.

At the time of enrollment, all patients underwent ophthalmic examinations including indirect ophthalmoscopy, non-contact intraocular pressure, fundus photography, BCVA (measured on ETDRS charts), axial length (IOL Master 500, Carl Zeiss Meditec, Jena, Germany), SD-OCT, OCTA, MP on the same day.

### Microperimetry

**Fig. 1:**
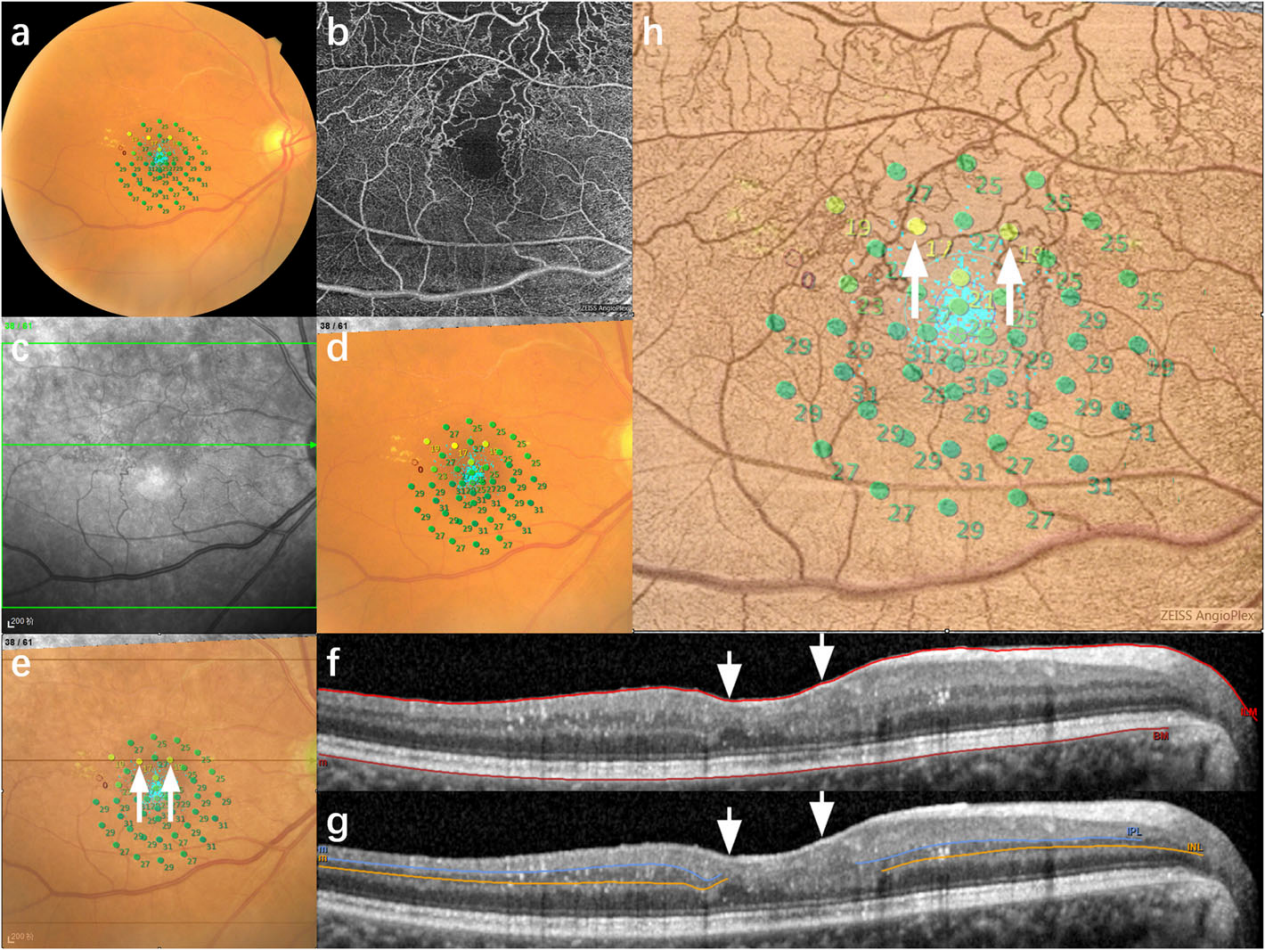
Multimodal images of a eye with BRVO. **a** Retinal sensitivity map of MP-3. The retinal sensitivity was evaluated at 45 stimulus points covering the central 12° (one at the center, 4 at 2°, 8 at 4°, 16 at 8°and 16 at 12°). **b** OCTA image(6 × 6 mm) of the entire retina centered on the fovea. **c** IR image of OCT. **d** MP-3 map image was registered to OCTA image by four shared anatomical landmarks. **e** MP-3 map image was registered to IR image and superimposed on IR image. **f and g** B-scan SD-OCT which was acquired along the line (**in “e”)** passing through two stimulus points (white long arrows ), demonstrated the existence of DRIL in the two point (white short arrows), with thickness of 242 μm (left) and 268 μm (right). **h** The registered MP-3 image was overlaid on the color-inverted OCTA image which demonstrated that the two points (white long arrows ) were located in NPA with sensitivity of 17dB (left) and 19dB (right)

MP (MP-3, Nidek Technologies, Gamagori, Japan) was performed by applying a grid of 45 stimulus points which covered the central 12° of the macula (Fig. 1a). The grid was divided into three circles that were within 4°and at 8° and 12°from the center. Goldman III–sized stimuli with a duration of 200ms and a dynamic range of 34dB ware presented against a white background of 31.4asb using a 4 − 2 (fast) staircase threshold strategy. Macular sensitivity was calculated as the average retinal sensitivity (RS) of the 45 stimuli points.

### Optical coherence tomography angiography

OCTA was performed with Cirrus HD-OCT 5000 (Carl Zeiss Meditec, Dublin, CA, USA) using the 3 × 3mm and 6 × 6mm scan pattern centered fovea (Fig. 1b). The vascular images of the entire retina, which spanned from the internal limiting membrane (ILM) and 70 μm above the retinal pigment epithelium (RPE), were automatically segmented.

### Optical coherence tomography

Dense macular volume scans covering 30°×25° of the macula centered on the fovea (Fig. 1c), and included 61 horizontal B-scans with an interval of 120 μm, were examined by the Spectralis SD-OCT (Heidelberg Engineering, Heidelberg, Germany).

### Data analysis

Infrared reflectance (IR) images obtained from OCT and OCTA images were matched with MP-3 map images using ImageJ (National Institutes of Health, http://imagej.nih.gov/ij/). Four shared anatomical landmarks were marked for image registration via affine transform [18, 19] (Fig. 1d). The images were overlaid to identify the microstructure and the perfusion status of the MP-3 measurement points (Fig. 1e-h).

The following items were evaluated on the OCT B-scans at the position corresponding to the stimulus points: DRIL, ELM disruption, ellipsoid zone (EZ) disruption, intraretinal cysts, hard exudates and outer retina layers disruption (defined as disruption of ELM or/and EZ) [20]. The retinal thickness (RT) of the stimulus points were measured from the ILM to the Bruch’s membrane [21] The retinal layer was first automatically distinguished using the built-in software; in case misidentifications occurred, manual corrections were then performed to obtain precise results.

The perfusion status of the stimulus points was determined via 6 × 6mm whole-retina vascular images. The foveal avascular zone (FAZ) was defined as the area within the innermost ring of the parafoveal capillary network. In cases the innermost ring was disrupted, the presumed FAZ was compensated by the innermost ring on en-face OCT [17]. The parafoveal NPA was defined as the capillary dropout area, not including the FAZ.

The microstructure and perfusion status of MP-3 measurement points were independently judged by two fully-trained retina doctors (PW and CL), who had no prior knowledge of the retinal sensitivity or any other eye conditions. When disagreements arose, the decision was arbitrated by a third experienced physician in retinal disease specialty after mutual discussion.

### Statistical analysis

The Kolmogorov-Smirnov test was used to assess normality. Continuous variables were expressed as mean ± standard deviations (SD) or median (interquartile range, IQR). The proportion of microstructure alterations and the average retinal sensitivity and thickness of the measurement points within NPA and the perfused area (PA) were calculated for each eye. Wilcoxon signed rank test was used to compare the proportion of microstructure alterations, and a paired samples t-test was used to compare the average retinal sensitivity and thickness. RS and RT of MP-3 measurement points in NPA were expressed as mean ± SD, and compared by generalized estimating equations with an exchangeable correlation matrix, patients as repeated subject variables, and microstructure alterations and circles of test grid (when comparing RT) as predictors factors. Generalized estimating equations was also used to determine the correlations between RS and RT. A *P*-value < 0.05 was considered statistically significant. All analyses were performed using IBM SPSS software (Version 21.0; IBM Corp., Armonk, NY, USA).

## Results

Thirteen patients (4 men and 9 women) were enrolled, with a mean age of 57.6 ± 7.7. The follow-up period ranged from 5 to 65 months. The subjects’ characteristics are shown in Table 1.

**Table 1:** Characteristics of subjects

Characteristics	*n* = 13
Age, year	57.6 ± 7.7
Gender(male: female), n	4:9
Affected eye(right: left), n	4:9
AL, mm	23.39 ± 0.55
IOP, mmHg	16.4 ± 2.2
Subtype of BRVO(Major BRVO: Macular BRVO), n	8: 5
Duration of follow-up, months	20 (5–65)
Duration of macular edema, months	8 (1–24)
BCVA,ETDRS letters	66.8 ± 8.0
Macular sensitivity, dB	24.9 ± 2.7
CRT, µm	242.5 ± 40.1
Baseline CRT, µm	389.0 (261.5)
Laser treatment, n	7
Anti-VEGF treatment, n	9
No laser or anti-VEGF treatment, n	1

Age, AL, IOP, BCVA, Macular sensitivity and CRT are expressed as the mean ± SD; Duration of follow-up and Duration of macular edema are expressed as medians (min–max); Baseline CRT is expressed as medians (IQR)

*AL* axial length, *IOP* intraocular pressure, *BCVA* best-corrected visual acuity, *CRT* central retinal thickness, *VEGF* vascular endothelial growth factor

Of the 585 MP-3 measurement points, there were 138 detected in NPA (40 within 4°, 53 at 8°, 45 at 12°), 400 (82 within 4°, 155 at 8°, 163 at 12°) in PA, and 47 in FAZ.

The average retinal sensitivity in NPA (18.2 ± 7.6dB) was significantly less than that in PA (27.4 ± 1.7dB, *P* = 0.001). The average retinal thickness in NPA (283.0 ± 43.2 μm) was significantly lower than that in PA (322.2 ± 22.6 μm, *P* = 0.003).

Table 2 shows the number of measurement points and eyes that present microstructure changes determined with SD-OCT B-scans and the proportion of microstructure changes in PA and NPA. In NPA, as many as 75 of 138 points showed DRIL, and the median of proportion was 64.3 (36.8)%, compared with 0.0 (1.5)% in PA, which was a significant difference (*P* = 0.002). The proportions of ELM disruption and EZ disruption were also significantly different between NPA and PA (*P* = 0043, *P* = 0.018). Disruption of the outer retinal layers existed in 28 points in NPA compared with 6 points in PA, and there was a significant difference between the proportions (*P* = 0.018). Moreover, cysts were more frequently observed in NPA, while the difference in proportion of cysts between NPA and PA was not statistically significant (*P* = 0.068).

**Table 2:** The relationship between perfusion status and microstructure alterations

Microstructure alteration	cases found in NPA (points/eyes), n	cases found in PA (points/eyes), n	Proportion in NPA, % (*n* = 13)	Proportion in PA, % (*n* = 13)	*P* value
DRIL	75 /12	5 /3	64.3 (36.8)	0.0 (1.5)	0.002
Outer retinal layers disruption	28 /7	6 /1	7.7 (20.9)	0.0 (0.0)	0.018
EZ disruption	22 /5	6 /1	0.0 (13.4)	0.0 (0.0)	0.043
ELM disruption	25 /7	5 /1	7.7 (20.9)	0.0 (0.0)	0.018
Both EZ and ELM disruption	19 /5	5 /1	0.0 (13.4)	0.0 (0.0)	0.043
Cysts	11 /4	0 /0	0.0 (20.6)	0.0 (0.0)	0.068
Hard exudates	2 /1	1 /1	0.0 (0.0)	0.0 (0.0)	0.655

Proportions of microstructural alteration are expressed as median (IQR)

*NPA* nonperfusion area, *PA* perfusion area, *DRIL* Disorganization of the retinal inner layers, *EZ* ellipsoid zone, *ELM* external limiting membrane

**Fig. 2:**
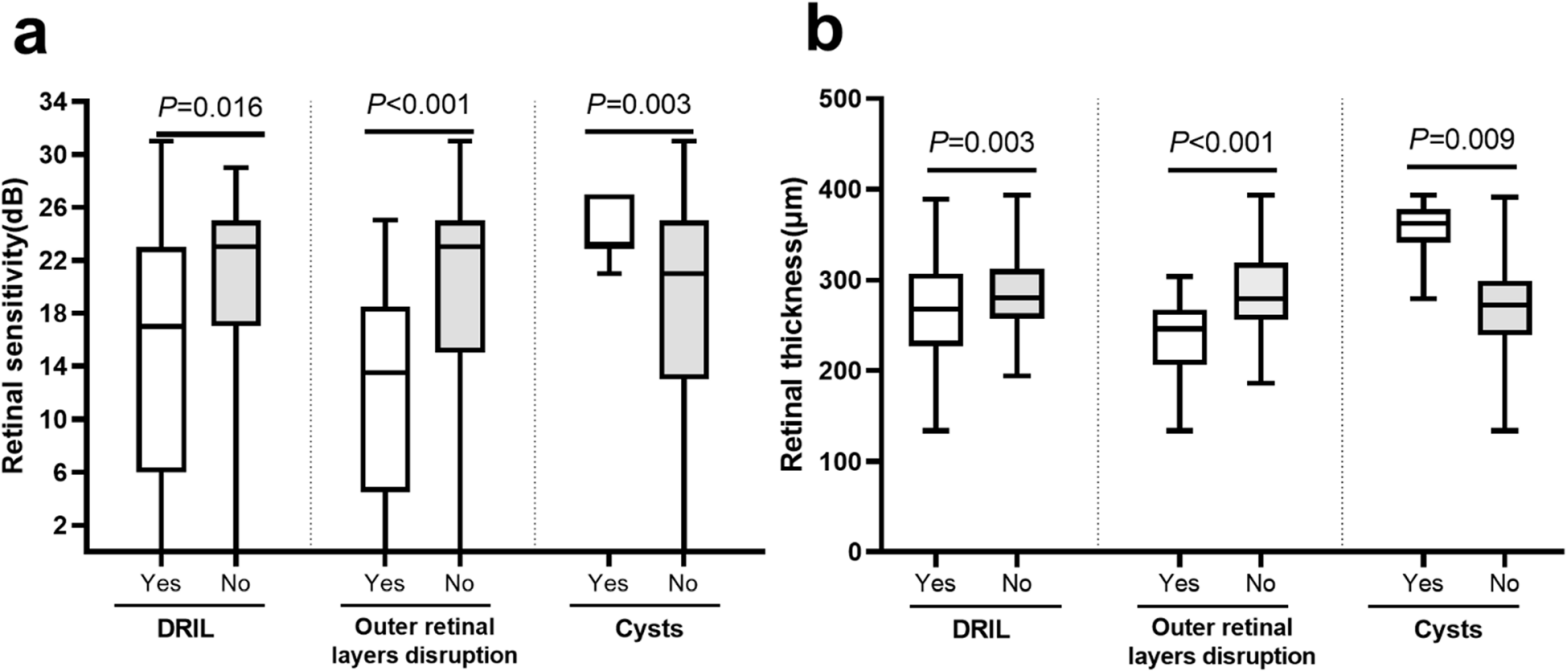
Comparison of retinal sensitivity (**a**) and retinal thickness (**b**) of MP-3 measurement points in the presence and absence of microstructure alterations in NPA

**Fig. 3:**
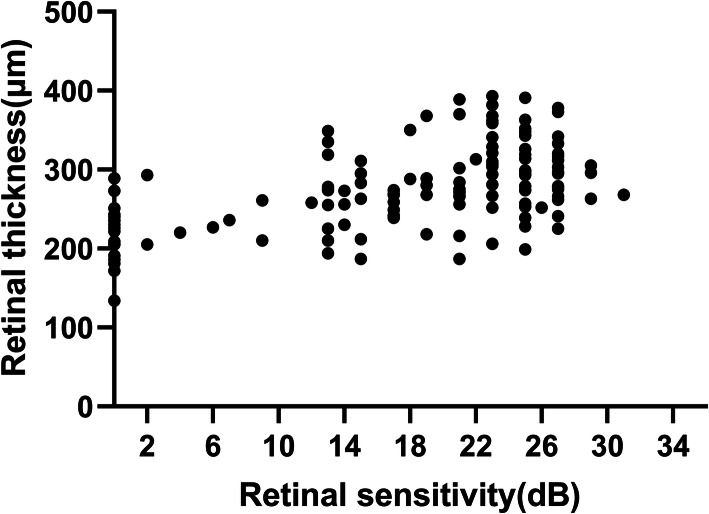
Scatter plot of retinal sensitivity and thickness in NPA

Among a total of 138 MP-3 measurement points in NPA the differences in RS (Fig. 2a) and RT (Fig. 2b) between the presence and absence of microstructure changes were analyzed. The RS of the points with DRIL (15.2 ± 10.1dB), and disrupted outer retinal layers (12.1 ± 8.3dB) were significantly lower than that of the points without DRIL (20.9 ± 7.2dB, *P* = 0.016), and with intact outer retinal layers (19.2 ± 9.0dB, *P* < 0.001). In contrast, the RS of the points located in cysts (24.3 ± 2.1dB) was significantly higher than that of the other points in NPA (17.2 ± 9.4 dB, *P* = 0.003). In NPA, the RT was significantly lower in the points that were present of DRIL (269.9 ± 55.9 μm), and outer retinal layers disruption (236.2 ± 40.6 μm), and absent of cysts (270.0 ± 47.5 μm) than those that were absent of DRIL (284.8 ± 45.5 μm, *P* = 0.003), and outer retinal layers disruption (287.0 ± 49.3 μm, *P* < 0.001), and present of cysts (353.5 ± 35.3 μm, *P* = 0.009). Furthermore, 1 dB increase of RS was correlated with 2.2 μm (95 % confidence interval, 1.71–2.7) increase in RT (*P* < 0.001) (Fig. 3).

**Fig. 4:**
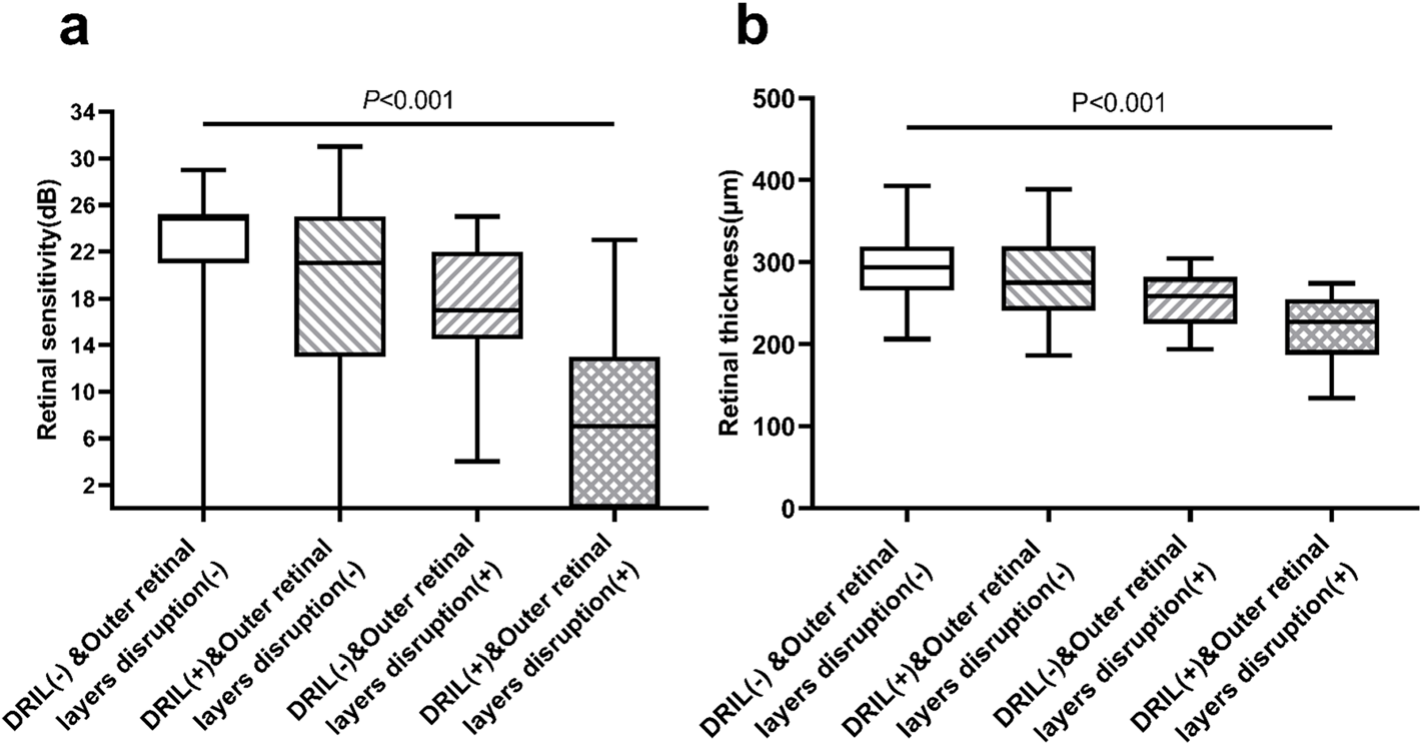
Comparison of retinal sensitivity (**a**) and retinal thickness (**b**) between status of inner and outer retinal layers. *n* = 50, 60, 13, 15, respectively

The points in NPA were divided into four groups according to status of the inner and outer retinal layers, as shown in Fig. 4. The mean RS in those groups were 21.7 ± 7.3dB, 17.2 ± 9.7dB, 17.6 ± 5.6dB, and 7.3 ± 7.3dB respectively, showing a significant difference (*P* < 0.001). The mean RT of the four groups were 292.7 ± 44.9 μm, 282.3 ± 52.6 μm, 254.7 ± 34.8 μm, and 220.1 ± 39.4 μm, respectively, with significant difference (*P* < 0.001). Pairwise comparison showed that the RS and RT in DRIL(-)&Outer retinal layers disruption(-) group were significantly higher than those in DRIL(+)&Outer retinal layers disruption(-) (*P* = 0.008, *P* = 0.009), DRIL(-)&Outer retinal layers disruption(+) (*P* < 0.001, *P* < 0.001), and DRIL(+)&Outer retinal layers disruption(+) (*P* < 0.001, *P* < 0.001). Furthermore, the RS and RT in DRIL(+)&Outer retinal layers disruption(+) group were significantly less than those in DRIL(+)&Outer retinal layers disruption(-) (*P* = 0.001, *P* = 0.039), DRIL(-)&Outer retinal layers disruption(+) (*P* = 0.001, *P* = 0.048). The RS and RT were not significantly different between DRIL(+)&Outer retinal layers disruption(-) and DRIL(-)&Outer retinal layers disruption(+) (*P* = 0.197, *P* = 0.418).

## Discussion

This study shows that the mean retinal sensitivity in NPA was 18dB compared with 27dB in PA, which demonstrates that the retinal sensitivity is significantly decreased in NPA. Previous studies reported a markedly decreased retinal sensitivity in areas within NPA [15, 16, 19]. Ota et al. [19] reported that retinal sensitivity within the NPA was substantially reduced, almost to an absolute scotoma (0.3 ± 1.3dB). However, the dynamic range (20dB) of MP-1 might be too small to correctly assess the retinal sensitivity in NPA, and judging the perfusion status using FFA might be affected by leaking dye. Adopting OCTA and MP-3, Tomiyasu et al. [15] recently reported that the mean retinal sensitivity in PA and NPA of eyes with RVO were 26dB and 18dB, respectively, which were similar to our results. We also confirmed that the mean thickness in NPA was lower than in PA, and the retinal sensitivity has a positive correlation with the retinal thickness in NPA [15].

DRIL is a SD-OCT feature that represents any boundaries between the ganglion cell–inner plexiform layer complex, inner nuclear layer, and outer plexiform layer cannot be identified [7]. Sun et al. [7] speculated that DRIL could represent the disorganization or destruction of cells within the inner retinal layers (bipolar, amacrine, or horizontal). It is possible that both mechanical [22] and vascular factors could be contributing to the pathogenesis of DRIL. Nicholson et al. [13] previously found that DRIL was a reliable predictor for macular nonperfusion areas in FFA in eyes with DR, that was present in 84.4 % of non-perfused retinas, and none in perfused retinas. Recently, by assessing the projection-resolved OCT angiography in eyes with DR, Onishi et al. [14] identified 85.7 % DRIL showing multilevel capillary nonperfusion, with superimposed flow impairment of the inner retinal capillaries on top of deep capillary plexus ischemia. In our study, DRIL was found in 75 of the 138 measurement points in NPA, while only 5 of the 400 points in PA. When NPA was identified by the whole retina layer, our study provided evidence that multilevel capillary ischemia plays an important role in the pathogenesis of DRIL. However, this hypothesis requires further study with OCTA to confirm the exact capillary layer that is responsible for DRIL.

We noted that in NPA, the retina with DRIL are thinner than that without DRIL, which was consistent with the findings of Nicholson et al. [13]. To date, several investigators have confirmed that the extent of DRIL is associated with reduced visual function in eyes with ME secondary to RVO [8–12]; most of them only evaluated the length of DRIL in an area of 1000 μm, but examination of a larger area of macula was recommend for more detailed and objective information [6]. To date, no study has examined the sensitivity of retina with DRIL in eyes with RVO, which may be able to provide a direct association between anatomy and function. We found that the retinal sensitivity was lower in the presence of DRIL. Previous investigators suggested that prolonged ischemia resulted in the atrophy of inner retinal layers [23], and before advanced irreversible atrophy took place, the ischemic retina was thicker and edematous [24]. These findings imply that DRIL was associated with more serious tissue degradation [23], and that functional damage and was likely to be present when a significant level of ischemia is reached.

Intraretinal cysts were more frequently located within NPA, although slightly below significance. MP-3 examination showed the function of the retina was better when cysts were discovered among NPA, which is similar to the findings of Tomiyasu et al. [15]. Studies have reported that cystoid spaces were colocalized within areas of capillary nonperfusion, as detected by OCTA [5, 25]. In addition, areas with severe capillary loss did not necessarily show significant cystoid edema [26]. In our study, edema was mild at all measurement points, which was unlikely to result in the further progression of the capillary dropout. It seems that a residual flow below the threshold can account for the reserved functions.

The majority of the literature reports that the extent of DRIL is a significant predictor of visual function in multivariable modelling [10, 11]. However, some of the literature reported that disruption in the outer retina was independently predictive of VA [12, 27]. Both the integrity of ELM and EZ were good parameters for assessing the degree of microstructural changes and functional recovery of the photoreceptor layer [28]. Scarinci et al. [29] reported that macular photoreceptor disruption on OCT in patients with DR corresponded to areas of capillary non-perfusion at the deep capillary plexus level. Our study examined the associations between disruptions of the outer retinal layers and perfusion status, which supported the hypothesis that ischemia plays an important role in photoreceptor disruption. Our results indicated that retina of nonperfusion area where the outer retinal layer was disrupted, suffered more serious functional damage. This was similar to the findings of Ota et al. [19], who found that the retina had an absolute scotoma, as detected by MP-1.

DRIL and EZ disruption were proved to affect BCVA in the ischemic state [6]. Our study further explored the difference under different statuses of the inner and outer retinal layers, indicating that the sensitivity and thickness of the retina were further substantially reduced when both layers were disrupted. Given these findings, we believe that the disruption of inner and outer retinal layers may be a sign of considerable function destruction within the nonperfused area, which represents great damage to signal production and transmission. Although the cause and effect cannot be inferred from this current cross-sectional design, we hypothesize the retinal function destruction is probably a consequence of more significant ischemia.

Our study had several limitations. Firstly, the sample size was small and the study had a cross-sectional design. Secondly, there were differences in the treatment regimens and follow-up periods. The interventions and duration of ME may influence the perfusion status or microstructure. In the current study, however, patients were evaluated after the complete or partial resolution of the ME. Further large prospective studies are necessary to elucidate the influencing factors and causal relationship of retinal function, perfusion status, and microstructure changes.

## Conclusions

In conclusion, macular microstructure alterations are associated with ischemia, especially in terms of DRIL. DRIL and outer retinal layers disruption are imaging features that have important implications for local retinal sensitivity in the ischemic areas, and where the microstructure of both inner and outer retinal layers is disrupted the function is further destructed.

## Data Availability

The data that support the findings of this study are available from the corresponding author upon reasonable request.

## Abbreviations

BRVO: Branch retinal vein occlusion
ME: Macular edema
VEGF: Vascular endothelial growth factor
RVO: Retinal vein occlusion
DRIL: Disorganization of the retinal inner layers
ELM: External limiting membrane
SD-OCT: Spectral domain-optical coherence tomography
MP: Microperimetry
OCTA: Optical coherence tomography angiography
NPA: Nonperfusion area
CRT: Central retinal thickness
BCVA: Best-corrected visual acuity
ETDRS: Early Treatment Diabetic Retinopathy Study
RS: retinal sensitivity
ILM: Internal limiting membrane
RPE: Retinal pigment epithelium
IR: Infrared reflectance
EZ: Ellipsoid zone
RT: Retinal thickness
FAZ: Foveal avascular zone
AL: Axial length
IOP: Intraocular pressure
PA: Perfused area
